# Actualización en insuficiencia mitral funcional: una revisión integral

**DOI:** 10.47487/apcyccv.v1i3.70

**Published:** 2020-09-30

**Authors:** René Ricardo Rodríguez Olivares, José María Carrasco Rueda, José Luis Añorga Ocmin, Carlos Lobato Jerí

**Affiliations:** 1 Servicio de cardiología clínica, Instituto Nacional Cardiovascular-INCOR. Lima, Perú. Lima Perú

**Keywords:** Insuficiencia de la válvula mitral, Insuficiencia cardíaca, Disfunción ventricular, Ecocardiografía, Mitral valve insufficiency, Heart failure, Ventricular disfunction, Echocardiography

## Abstract

Los avances en el manejo de la insuficiencia mitral (IM) funcional en las últimas décadas, hacen que conocerlos sea de suma importancia para el cardiólogo clínico, debido a la prevalencia cada vez mayor en pacientes mayores de 50 años. Esta revisión tiene como objetivo dar a conocer de una manera integral las bases fisiopatológicas de la IM para diferenciar sus mecanismos y correlacionarlos con los hallazgos clínicos, hemodinámicos y de imágenes que valoran la severidad de la enfermedad. Estos datos son relevantes para la adecuada selección del paciente y del momento oportuno de las intervenciones terapéuticas, lo cual involucra a un equipo multidisciplinario en la toma de decisiones con el fin de lograr el mayor beneficio dentro de las opciones de tratamiento de esta patología.

## Abstract (en)

In recent decades, the advances in the management of functional mitral regurgitation (MR), makes knowledge of this disease extremely important to the clinical cardiologist, as it has an increasing prevalence in patients older than 50 years. The objective of this article is to review the pathophysiological bases of MR, in order to differentiate its mechanism, and to correlate them with the clinical, hemodynamic, and imaging findings that assess the severity of the disease. These data are relevant for the adequate selection of the patient and the opportune moment of the therapeutic interventions, which involves a multidisciplinary team in decision-making to achieve the greatest benefit within the treatment options of this pathology.

El aparato valvular mitral es una estructura funcional, compleja y dinámica, cuya alteración puede cambiar significativamente el funcionamiento del sistema cardiovascular ^1^. A inicios de este milenio la insuficiencia mitral (IM) afectaba aproximadamente a 2,5 millones de habitantes en Estados Unidos y Europa, siendo la valvulopatía más frecuente en la práctica clínica cardiológica ^2^^,^^3^. Tiene una prevalencia estimada de 1,7% (Estados Unidos), siendo mayor en pacientes con más de 75 años (9,3%) y menor a 1% en menores de 55 años ^2^^,^^4^.

Se reporta una prevalencia de insuficiencia mitral funcional (IMF) asociada a cardiopatía isquémica de 7500 a 9000 casos por millón de habitantes, y en relación a la de etiología no isquémica, de 16250 casos por millón de habitantes, representando en occidente la segunda indicación más frecuente de cirugía cardiaca ^4^. Está presente en el 25% de pacientes posinfarto y en el 50% de pacientes con falla cardiaca, disfunción ventricular izquierda y cardiomiopatías ^2^^,^^4^. Generalmente tiene una presentación leve (47%) a moderada (17%), la cual progresa a estadios severos (9%), asociándose con mayor morbimortalidad ^5^.

El objetivo de este artículo es ofrecer una revisión de la fisiopatología de la IMF, enfocada en explicar diferentes opciones diagnósticas y terapéuticas.

## Definición

El aparato valvular mitral está conformado por el anillo mitral, las valvas anterior y posterior, las cuerdas tendinosas, los músculos papilares anterolateral y posteromedial y el miocardio del ventrículo izquierdo (VI) que rodea los músculos papilares. Cualquier disfunción de estas estructuras puede causar IM ^1^^,^^3^.

Mecanismo y causa de IM no son sinónimos; cuando nos referimos a las causas hablamos de enfermedades que comprometan el aparato valvular mitral y patologías que producen remodelado del VI ^6^. Al hablar de mecanismos tenemos dos tipos: orgánica y funcional; siendo esta última la más común. La IM orgánica o primaria se relaciona con una alteración estructural propia, debido a lesión de alguno de los componentes valvulares, y la IMF (secundaria) con una restricción en su coaptación debido a la disfunción ventricular izquierda subyacente, dilatación del anillo mitral, o ambas, en velos valvulares normales (o pseudonormales); siendo su presentación más clásica la restricción de la motilidad de las valvas en sístole ^1^^,^^6^.

## Fisiopatología

Anteriormente se planteaba que la causa de la IMF era la presencia aislada de una falla de coaptación valvar, manifestada como un orificio regurgitante anatómico, pero solo un pequeño porcentaje de pacientes presentaban imágenes de orificio regurgitante y, en su lugar, la mayoría poseía un fenómeno de tracción (*tethering*) ejercido sobre las valvas, considerándose este un marcador de IMF ^6^^,^^7^.

**Figura 1.**
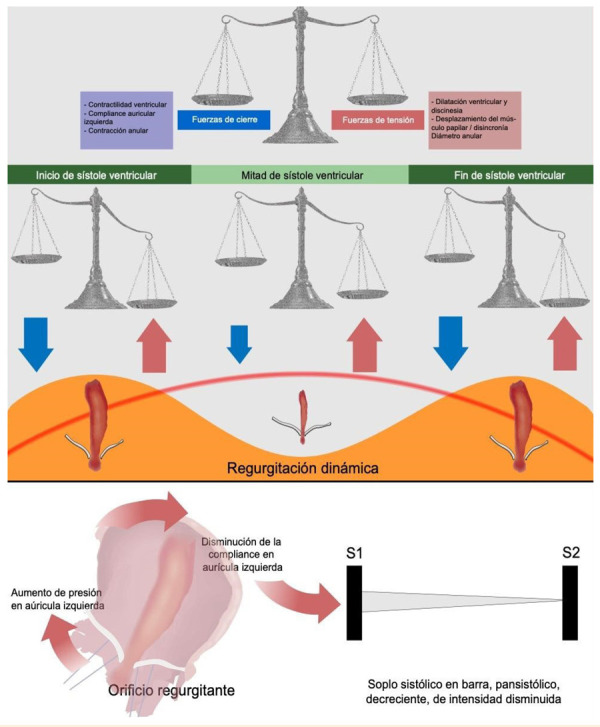
Fisiopatologia del mecanismo de la insuficiencia mitral funcional

 El *tethering* valvular es un desequilibrio entre las fuerzas de cierre y las de tracción valvular, ya sea por un incremento de las segundas (a nivel muscular papilar o anular), o una disminución de las primeras, por disfunción ventricular izquierda ^8^^)^ **(**Figura 1**).** La forma más frecuentemente de IMF es la insuficiencia mitral isquémica (IMI) producida tras un infarto de miocardio, debido a un desbalance entre los componentes del aparato subvalvular mitral (incremento en las fuerzas de anclaje y reducción en las fuerzas de cierre) y una estructura valvular normal ^9^^,^^10^. La IMI tiene como causa la alteración de la geometría ventricular, la isquemia debida a un infarto previo o, con menos frecuencia, a la isquemia transitoria (con mejoría de la insuficiencia mitral una vez resuelto el evento) ^11^.

A. En condiciones fisiologicas existe un equilibrio entre las fuerzas de cierre y traccion valvular mitral. Cuando se produce una deficiencia de los primeros, o un exceso de los segundos, se genera la regurgitacion. B. Dinamica del flujo regurgitante (curva con fondo naranja) a traves de las fases de la sistole ventricular. El desequilibrio entre las fuerzas de cierre y traccion varia en cada una de ellas debido a la variacion de la presion intraventricular (curva roja) a traves de ellas, siendo esta mayor a mitad de la sistole, haciendo que la caida de las fuerzas de cierre se corrija parcialmente y, por consiguiente, ocurra una reduccion en el flujo regurgitante. C. Cualidad del soplo en la insuficiencia mitral secundaria. Dado que la fisiopatologia como particularidad un especial incremento en las presiones de llenado de la auricula izquierda y, por ende, una reduccion progresiva de la compliance de la misma, la energía generada por el flujo regurgitante no puede traducirse en energia cinetica a traves del orificio regurgitante, por lo que, mas bien, se traduce en energia potencial. Esto, por un lado, incrementa la presion auricular izquierda y, por otro, reduce la intensidad del soplo, a comparacion de la insuficiencia primaria, conservando sus demas caracteristicas auscultatorias.

Los datos más resaltantes en IMI se pueden englobar de la siguiente manera ^9^^,^^12^:

● Insuficiencia mitral que aparece durante la primera semana luego de un infarto de miocardio.

● Movimiento anormal de uno o más segmentos ventriculares. 

● Enfermedad coronaria significativa.

● Velos valvulares y aparato subvalvular normales. 

**Figura 2:**
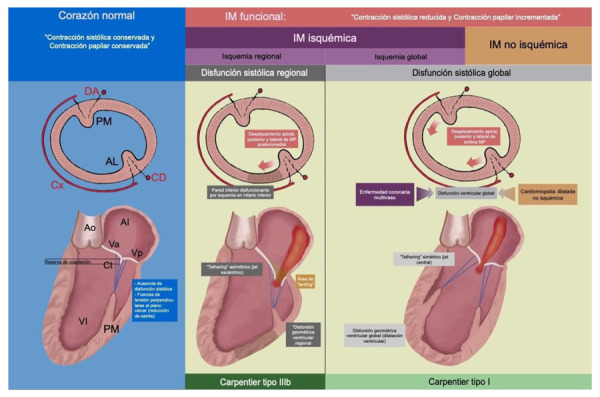
Mecanismos y clasificacion de la insuficiencia mitral funcional

A raíz de los conceptos teóricos descritos líneas arriba, se han planteado varios mecanismos prácticos de disfunción **(**Figura 2**).**

Se muestra la clasificacion y los mecanismos asociados a insuficiencia mitral secundaria, tomando como referencia etiologías tipicas. IM: insuficiencia mitral. DA: arteria descendente anterior. Cx: arteria circunfleja. CD: coronaria derecha. PM: musculo papilar posteromedial. AL: musculo papilar anterolateral. Ao: aorta. AI: auricula izquierda. VI: ventriculo izquierdo. Va: valva anterior mitral. Vp: valva posterior mitral. Ct: cuerdas tendineas.

### 1. Disfunción intrínseca de los músculos papilares

Diversos estudios experimentales en animales han demostrado que la disfunción de la musculatura papilar *per se* (inducida por infarto) no condiciona la IM, pudiendo, inclusive, prevenirla, por lo que actualmente se recomienda no utilizar el término de disfunción intrínseca de músculos papilares como parte del proceso fisiopatológico de esta enfermedad ^13^.

### 2. Disfunción ventricular izquierda

En un estudio experimental en animales, al inducir farmacológicamente disfunción sistólica del VI, limitando su remodelamiento (dilatación) por constricción pericárdica, se observó que la IMF que generaba era de grado leve. Por el contrario, si se eliminaba la constricción pericárdica y se permitía el remodelado ventricular el grado de IMF era severo, concluyéndose que la disfunción ventricular aislada no ocasiona IM significativa ^14^. Otro estudio realizado en pacientes con oclusión coronaria aguda y disfunción ventricular transitoria, observó que durante el periodo de oclusión coronaria, acompañada de trastorno extenso de motilidad, se presentó IM significativa solo en 1 de los 83 pacientes analizados ^15^.

Por lo anterior, se concluye que la disfunción sistólica ventricular izquierda aislada (sin remodelamiento) no es capaz de producir el fenómeno de *tethering* subyacente a la IMF significativa ^7^^,^^15^.

### 3. Integridad geométrica: músculos papilares y anillo mitral

Los mayores determinantes de la IM en pacientes con disfunción ventricular izquierda son: el *tenting* valvular sistólico, la contracción anular, el volumen telediastólico indexado y el desplazamiento posterior de los músculos papilares; siendo la fracción de eyección y el remodelamiento del VI, variables predictoras no significativas de IM ^7^.

Dichos resultados han sido confirmados en estudios *in vivo*^16^ y se han evidenciado en un modelo *in vitro*^17^, que permitía la movilización (malposición) de los músculos papilares, la ampliación o reducción del anillo y la modificación de la presión transmitral. En dicho estudio se observó que la presencia de un anillo con diámetro normal asociado a malposición de músculos papilares incrementraba el grado de IM, sin evidencia de que la disfunción de VI aislada aumente la IM, a menos que existiera un desplazamiento apical del músculo papilar. Asimismo, la IM producida por un anillo dilatado empeoraba con la disfunción geométrica del músculo papilar ^7^^,^^17^.

### 4. La integridad de la válvula mitral

La indemnidad es parte de la definición de IMF, pero reconstrucciones tridimensionales han revelado lo contrario ^18^^,^^19^. Mediante técnicas de medición de las áreas de superficie valvular y de cierre, se halló que en pacientes normales existe una redundancia entre el tejido valvular disponible con respecto a la superficie mínima necesaria para ocluir el orificio valvular. En pacientes con IMF significativa, los velos son desplazados por debajo del anillo causando un incremento del área de superficie valvular y, proporcionalmente, una mayor área de cierre. Por otro lado, en pacientes con disfunción ventricular izquierda sin evidencia de regurgitación mitral, la redundancia proporcional se mantiene como en individuos normales. Esto explicaría por qué ante disfunciones severas de VI podría no desarrollarse IM significativa, al conservarse la competencia valvular por la redundancia apropiada de esta ^17^.

Por lo tanto, podemos plantear que un evento disfuncionante de naturaleza isquémica o no, genera remodelado ventricular y anular con la aparición de *tethering* patológico. La IM ocurriría cuando la expansión es inadecuada o el remodelado es muy extremo; sin embargo, los cambios de adaptación valvular conllevan un comportamiento anormal de la misma, lo que incrementa su rigidez con el tiempo, determinando una naturaleza heterogénea de la patología ^4^^,^^8^. Finalmente, tras la aparición de IMF, la sobrecarga de volumen estimulará el empeoramiento del remodelado mediante el incremento del estrés parietal que promueve hipertrofia excéntrica del VI ^7^^,^^18^.

Cabe agregar la existencia de escenarios patológicos que, sin la presencia de disfunción sistólica evidente, llevan al desarrollo de IMF. Tal es el caso de la fibrilación auricular (por dilatación auricular izquierda), la estenosis aórtica (por aumento de la gradiente de presiones auriculo-ventriculares izquierdas) o la rigidez aórtica (a través del aumento de la poscarga). Esto se debe a que las presiones de llenado del VI que se oponen al cierre valvular, generan y empeoran el *tenting* valvular sistólico mitral, asociándose, además, a disfunción longitudinal oculta ^20^.

## Diagnóstico

### a. Historia clínica

La sintomatología es variable y su presentación depende de la gravedad de la regurgitación, el tiempo de evolución, así como de la actividad física del paciente ^1^^,^^2^. Tres mecanismos pueden explicarla: a) Aumento de la presión en la aurícula izquierda y congestión pulmonar, caracterizados por disnea de esfuerzo, disnea paroxística nocturna y ortopnea; b) Disminución del gasto cardíaco que produce fatigabilidad, adelgazamiento y c) Hipertensión pulmonar, derivando en insuficiencia ventricular derecha y síntomas de congestión sistémica ^1^.

La mayoría de pacientes con IM crónica significativa desarrollan síntomas de falla ventricular izquierda aproximadamente a los 10 años del diagnóstico, a diferencia de los cuadros agudos que pueden debutar con edema pulmonar y síntomas de insuficiencia cardiaca congestiva. Los pacientes con IM crónica responden bien al tratamiento médico de inicio, pero la presencia de insuficiencia ventricular izquierda o hipertensión pulmonar severa, traducen un pronóstico relativamente malo ^1^^,^^2^.

### b. Examen físico

Es factible observar un latido torácico diagonal y, en caso de dilatación del ventrículo derecho, un latido sagital. Habitualmente, a la palpación, el choque de punta es hiperdinámico y está desplazado hacia abajo y hacia la izquierda ^2^. A la auscultación, el primer ruido puede estar normal o alterado; el segundo ruido generalmente está ausente debido a la sobreposición del soplo regurgitante, en tanto que la presencia de tercer ruido suele ser expresión de gravedad de la lesión valvular. El soplo de la IM se ausculta a nivel del ápex y suele ser en barra, pansistólico y decreciente **(**Figura 2**)**; puede irradiarse a todo el precordio, el dorso y, especialmente, a la axila. Cuando el soplo es de baja intensidad se recomienda efectuar la auscultación en decúbito lateral izquierdo (maniobra de Pachón) ^2^^,^^3^. Es posible encontrar signos de congestión venosa sistémica como ingurgitación yugular, hepatomegalia y edemas en los miembros inferiores en presencia de insuficiencia ventricular derecha ^2^.

### c. Ecocardiografía

Permite evaluar el aparato valvular mitral, las dimensiones de cámaras cardíacas, la función biventricular, el grado de insuficiencia y las valvulopatías asociadas. En caso de mala ventana e información necesaria para manejo invasivo se complementa el estudio con la ecocardiografía transesofágica ^1^.

El modo 3D es considerado el *gold standard* para evaluar las dimensiones y la dinámica de la válvula mitral. Su uso en el estudio del jet regurgitante es superior al modo 2D al evaluar el área valvular, el origen del jet y la dimensión de la vena contracta especialmente en presencia de chorros múltiples o excéntricos ^1^^,^^21^. Según la valoración ecocardiográfica, la IMF presenta dos patrones bajo la clasificación de Carpentier ^1^^,^^7^:

Tipo I. Movimiento y posición valvular normales, se da por defecto de coaptación. Causas: dilatación auricular izquierda, estenosis aórtica, y cardiomiopatía hipertrófica obstructiva.

Tipo IIIb. Movimiento restrictivo sistólico de los velos por remodelamiento ventricular izquierdo. Causado por cardiomiopatía isquémica (*tethering* asimétrico) o dilatación ventricular (*tethering* simétrico).

Para la evaluación de la severidad se dispone de las siguientes herramientas ecocardiográficas **(**Tabla 1**)**:

● Métodos cualitativos: evaluación del chorro regurgitante y la duración e intensidad de la señal de regurgitación realizados a través del modo *doppler* y *doppler* color. Un jet > 40% del área de la aurícula izquierda indica la presencia de IM severa. Estos métodos no son precisos en jets excéntricos y varían según el estado hemodinámico ^21^.

● Métodos semicuantitativos: incluyen la medición de la vena contracta (> 7 mm), valoración del patrón de llenado de VI (velocidad máxima de onda E > 120 cm/s) y el flujo de venas pulmonares (el cual es normalmente anterógrado con un flujo retrógrado durante la sístole auricular, a mayor severidad mayor disminución del componente sistólico e inversión del flujo sistólico) ^21^.

● Métodos cuantitativos: medición del área de orificio de regurgitación basado en el área de la zona de isovelocidad proximal (PISA). El área de orificio regurgitante efectivo (EROA), basado en la ecuación de continuidad, permite valorar la severidad de la IM con un punto de corte de 0,4 cm^2^. También existen otros valores como la medición del volumen regurgitante (≥ 60 mL) o de la fracción regurgitante (≥ 50%) ^(^^21^.

**Tabla 1:** Criterios ecocardiográficos de severidad en pacientes con insuficiencia mitral funcional

Evaluación ecocardiográfica del grado de severidad de la IMF					
Cualitativa		Semicuantitativa		Cuantitativa	
Área del jet	Central> 50% de AI o muy excentrico	Vena contracta	≥7 (>8 en biplano)	EORA PISA	≥ 0,4 cm2
Flujo convergente	Largo a traves de la sistole	Flujo vena pulmonar	No flujo sistolico o sistolico reverso	Volumen regurgitante	≥ 60
Jet (doppler continuo	Denso, holosistolico, triangular.	Velocidad de flujo mitral	Onda E dominante > 120 cm/s	Fracción regurgitante	≥ 50%
				Área vena contracta 3D	> 0,41
Morfología		Tenting severo y pobre coaptacion valvular. Dilatacion de AI y VI en etapas cronicas.		

AI: aurícula izquierda. EORA: área del orificio regurgitante efectivo. PISA: área de la zona de isovelocidad proximal. VI: ventrículo izquierdo. Adaptado de Zoghbi WA, et al. ^(21)^.

La ecocardiografía de estrés es de utilidad en pacientes con síntomas desproporcionados respecto a la severidad de la IM, o a fin de determinar la reserva ventricular contráctil. Un aumento del EROA ≥ 0,13 cm^2^ y/o una presión arterial pulmonar >60 mmHg al esfuerzo, se asocian con peor pronóstico ^1^.

A nivel miocárdico se puede apreciar el remodelado regional que desplaza los músculos papilares, traccionando las valvas y produciendo una forma de «tienda» a nivel de las valvas (*tenting*), debido al anclaje anormal de las cuerdas (*tethering*), sea simétrico o asimétrico. La evaluación ecocardiográfica de estos fenómenos es importante y se ha correlacionado con la gravedad y etiología de la IM ^22^^,^^23^.

### d. Cateterismo cardiaco

Se utiliza para evaluar el estado hemodinámico y en la evaluación de las arterias coronarias. Una gran onda «V» en el registro de cuña pulmonar sugiere IM, pero su ausencia no la excluye. Además, podemos estimar la presencia de hipertensión pulmonar, tanto poscapilar aislada como mixta. Esta última sugiere enfermedad vascular pulmonar y alto riesgo de falla derecha tras la cirugía. De ahí la importancia de la adecuada medición de dichas presiones para optimizar terapia con vasodilatadores y diuréticos, de ser necesario ^1^.

### e. Resonancia magnética nuclear (RMN)

Considerada el *gold standard* en la evaluación funcional y dimensional de las cámaras cardiacas ^1^. Puede proveer una evaluación precisa de la disfunción miocárdica, motilidad segmentaria, fibrosis o grado de severidad, y evaluar el diámetro telediastólico del VI, sobre todo en el contexto de remodelamiento esférico apical y medioventricular de dicha cámara ^3^^,^^24^. Además, es superior a la ecocardiografía 2D y 3D para la caracterización de la disfunción del anillo mitral ^25^.

El uso de captación retardada de gadolinio y mapeo T1 permiten identificar fibrosis de la pared posterior del VI y de los músculos papilares, capaces de predecir eventos arrítmicos en IM degenerativa, pero aún está en estudio su uso en IMF ^26^^,^^27^.

## Clasificación

De acuerdo con la etiología y los mecanismos subyacentes, se ha clasificado a la IMF según su estructura, grado de coaptación y cierre valvular **(**Tabla 2**)**^28^.

**Tabla 2:** Características y diferencias de los fenotipos de insuficiencia mitral

Características	Insuficiencia mitral funcional	
	Disfunción regional VI	Disfunción global del VI
Etiología	Infarto de miocardio inferior	Miocardiopatía no isquémica, infartos de miocardio múltiples o anterior extenso.
Remodelado de VI	Principalmente pared inferior	Dilatacion global con incremento esfericidad.
Remodelado de AI	Variable	Generalmente severo
Anillo	Leve o sin dilatacion, menos dinamico.	Dilatado, aplanado, no dinamico
Morfologia de la valva:		
Engrosamiento	No / leve	No / leve
Prolapso o flail	No	No
Calcificacion	No / leve	No / leve
Patron de tethering	Asimetrico	Simetrico
Tenting sistolico	Aumentado	Marcado incremento
Distancia del musculo papilar	Mayor distancia fibrosa posterior papilar-intervalvular	Mayor distancia muscular interpapilar
Direccion del chorro de Insuficiencia mitral	Posterior	Generalmente central
Onda de doppler continuo	La densidad generalmente es uniforme en toda la sistole.	Patron bifasico, con aumento de la densidad en el flujo sistolico temprano y tardio, y dropout mediastolica
PISA	A menudo no hemisferico	A menudo no hemisferico; puede ser bifasico

VI: ventriculo izquierdo. AI: auricula izquierda. PISA: area de superficie de isovelocidad proximal. Adaptado de Bonow RO, *et al.* (28).

**IM por *tethering* asimétrico.** Considerada como la IM isquémica clásica, relacionada al infarto de cara inferolateral. Produce remodelamiento progresivo y un aumento del área de *tenting* valvular (leve a moderado). En la evaluación ecocardiográfica se evidencia un jet regurgitante posterior por restricción del movimiento del velo anterior. Estos pacientes presentan pobre respuesta al tratamiento médico, con indicación frecuente de manejo quirúrgico ^1^^,^^8^^,^^28^.

**IM por *tethering* simétrico.** Relacionado con infarto anterior extenso o múltiple y a cardiomiopatía no isquémica. Presentan un remodelamiento esférico excéntrico y *tethering* bivalvar evidenciado como un jet regurgitante central debido a la disfunción global de la motilidad ventricular. Estos pacientes tienen buena respuesta al tratamiento médico y/o terapia de resincronización cardiaca ^8^^,^^28^.

IM por dilatación anular. Propia de pacientes con fibrilación auricular. Es causada por remodelamiento auricular y alargamiento del anillo mitral, se manifiesta como un jet regurgitante central sin presencia de coaptación central de los velos ni *tethering* significativo ^3^^,^^4^. El retorno al ritmo sinusal en estos pacientes ha demostrado revertir el remodelamiento auricular izquierdo, reducir el tamaño anular y disminuir la severidad de la IM ^29^.

Cabe resaltar que la dilatación del anillo mitral aumenta la severidad de la IM si está asociada a *tethering*^3^^,^^8^.

## Tratamiento

### a. Tratamiento médico

Al inicio debe basarse en los parámetros terapéuticos establecidos según las guías de práctica clínica ^23^^,^^30^, su beneficio se establece por la modulación de los cambios fibróticos en la válvula mitral, regulación neurohormonal y reducción de la masa del VI ^30^.

Los inhibidores de enzima convertidora de angiotensina, diuréticos y beta bloqueantes son el pilar terapéutico y están indicados en todo paciente con disfunción ventricular izquierda e IMF. Un estudio observacional encontró que el uso de carvedilol en pacientes con insuficiencia cardiaca mejoraba la función sistólica al cabo de 6 a 12 meses, con disminución de la severidad de la IMF ^31^. En otro ensayo clínico, el carvedilol demostró reducir la masa ventricular y el remodelado esférico en pacientes con cardiomiopatía dilatada con mejoría de la función cardiaca. Por ello, se plantea que estos medicamentos, al impedir y revertir el progreso del remodelado ventricular, pueden reducir el grado de severidad de la IMF ^23^^,^^32^.

El uso combinado de fármacos para insuficiencia cardiaca ha demostrado una mejora hemodinámica significativa en pacientes con IMF severa, tal como lo reporta un ensayo clínico donde el uso de digoxina, diuréticos, IECA y metoprolol evidenció una reducción del volumen telesistólico y telediastólico del VI con mejoría de la IMF; sin embargo, no hubo reducción significativa en mortalidad y readmisión ^33^.

En el estudio PRIME ^34^, el uso de sacubitril/valsartán demostró reducción del área del orificio regurgitante efectivo en comparación a solo valsartán en pacientes con falla cardíaca con fracción de eyección reducida y regurgitación mitral funcional.

En otro estudio, el uso de digoxina y diuréticos, con dosis progresivas de lisinopril y dinitrato de isosorbide, demostró una disminución del grado de severidad de la IM en el 40% de los pacientes, una mejoría en la función sistólica del VI y disminución de las dimensiones telediastólicas del VI en pacientes que respondieron al tratamiento ^35^.

### b. Terapia de resincronización cardiaca (TRC)

Demostró ser beneficiosa en pacientes con IMF e insuficiencia cardiaca con tratamiento médico óptimo, gracias a su capacidad para mejorar las fuerzas de cierre valvular, reducir las fuerzas de *tethering*, reformar la función y geometría anular y corregir la regurgitación mitral diastólica ^1^. Su uso está recomendado, previo a la intervención quirúrgica o percutánea, en pacientes con ritmo sinusal, disnea NYHA II a VI (a pesar de tratamiento médico óptimo), fracción de eyección de VI <35% y bloqueo completo de rama izquierda (BRI) con un QRS > 150 ms. También puede ser útil en pacientes con QRS >150 ms y BRI ausente y en aquellos con BRI y QRS 120 a 149 ms ^6^.

El estudio MIRACLE ^36^ evaluó el uso de TRC en pacientes con BRI y QRS > 130 ms; se observó una reducción del volumen telesistólico y telediastólico del VI; mejoría de la función cardiaca y reducción del área del jet regurgitante mitral. En otros estudios de similares características (MUSTIC ^37^ y CARE-HF ^38^), la TRC demostró una reducción significativa de la severdidad en el 40 a 50% de pacientes, asociado a una mejoría de las dimensiones ventriculares, retraso interventricular, volumen telesistólico, función sistólica y síntomas luego de 3 a 6 meses de tratamiento ^37^^;^^38^.

Adicionalmente, se ha observado un incremento inmediato de la función sistólica y mejoría de la disincronía posterior al implante de TRC, en un 50% de casos, así como un remodelamiento reverso ventricular a largo plazo (12 meses) ^39^. Sin embargo, la persistencia de severidad en la IMF a los 3 - 6 meses posterior a TRC es un factor de mal pronóstico asociado con mayor presencia de eventos arrítmicos y menor remodelamiento inverso ^1^^,^^40^.

Un ensayo clínico aleatorizado observó que aquellos pacientes que respondieron al tratamiento hasta tres meses posteriores al implante de TRC, no requirieron de ningún tipo de intervención adicional a largo plazo; por lo que los autores proponen el retraso de la intervención quirúrgica hasta el tercer mes de seguimiento debido a la posibilidad de respuesta terapéutica ^41^.

### c. Intervención percutánea

Existen diferentes técnicas de abordaje, como la anuloplastía, el reemplazo valvular mitral transcateter y la reparación borde a borde ^42^^,^^43^.

El dispositivo de anuloplastía directa (Cardioband TM) es un abordaje prometedor que reproduce parcialmente al método quirúrgico y se utiliza en pacientes con IM asociada a dilatación de anillo mitral. Su uso ha reportado una reducción clínica significativa del grado de severidad de las dimensiones anulares y un aumento del remodelamiento reverso ^42^.

El abordaje por anuloplastia indirecta (Carillon) es un método sencillo y reproducible que se realiza a través de la colocación de un dispositivo en el seno coronario; debido a su proximidad con el anillo mitral. Lamentablemente y a pesar de su evidencia clínica positiva, este método tiene sus desventajas debido a la variabilidad del alineamiento del plano anular y el seno coronario, así como el riesgo de obstrucción de la arteria circunfleja ^42^^,^^43^.

En la última década, se han desarrollado técnicas de reemplazo valvular mitral percutáneo (Cardiovalve, Tendyne) con escaso progreso debido a la compleja anatomía de la válvula mitral y la variabilidad anatómica. A pesar de ello, hay evidencia de experiencia clínica con buenos resultados sobre su uso en determinados pacientes con alto riesgo quirúrgico ^42^.

El MitraClip es actualmente el tipo de abordaje más utilizado en la IMF, como alternativa a la intervención quirúrgica; cuyo objetivo es desarrollar un procedimiento de bajo riesgo capaz de reducir la severidad de la IM y asegurar una mejoría clínica significativa. Consta de una técnica de reparo percutáneo utilizando dispositivos de agarre para aproximar los bordes de los velos mitrales. Su uso está aprobado por la FDA para pacientes con IMF e insuficiencia cardiaca en tratamiento óptimo que posean contraindicación quirúrgica ^42^^,^^43^**(**Tabla 3**)**. A la fecha, existen tres estudios que han evaluado su empleo ^43^^-^^46^:

**Tabla 3:** Indicaciones, contraindicaciones y factores favorables para el uso de MitraClip en insuficiencia mitral funcional o secundaria

Indicación	Contraindicación
	Pacientes que no puedan tolerar el uso de anticoagulantes periprocedimiento.
Pacientes con IM moderada a severa, insuficiencia cardiaca refractaria a tratamiento medico optimo e indicación prohibitiva quirurgica.	Endocarditis activa valvular mitral.
Pacientes con comorbilidades que no impiden el beneficio esperado de la reduccion de la regurgitacion mitral.	Enfermedad reumatica valvular.
	Presencia de coagulos intracardiacos.
	Trombosis venosa femoral o de la vena cava inferior.
	FEVI <20% y/o VDFVI >70 mm
Factores favorables	
Calcificacion minima o ausente.	
Gradiente transmitral < 4 mm.	
Patologia valvular no comisural.	
Profundidad de coaptacion <11 mm y longitud de coaptacion > 2mm.	
Longitud de la zona de agarre > 10 mm.	
FEVI >25% y VDFVI ≤ 55 mm.	

FEVI: fracción de eyección de ventrículo izquierdo: VDFVI: volumen diastólico final del ventrículo izquierdo.

Adaptado de Gössl M, Sorajja P. MitraClip patient selection: inclusion and exclusion criteria for optimal outcomes. Ann Cardiothorac Surg. 2018;7(6):771-775. DOI: 10.21037/acs.2018.08.04

El estudio EVEREST II (Endovascular edge to edge Repair) ^45^, es el primero que comparó el tratamiento quirúrgico convencional y la intervención percutánea en pacientes con bajo riesgo quirúrgico. Los datos revelaron que, a pesar de ser una intervención percutánea relativamente segura, era menos efectiva que la cirugía, debido a poseer menor tiempo libre de muerte, cirugía valvular, reintervención o presencia de IM moderada a severa. Sin embargo, un subanálisis del estudio demostró el potencial beneficio de la intervención percutánea en pacientes mayores de 70 años.

Basados en los resultados de este estudio se desarrollaron otros dos ensayos clínicos sobre el uso de MitraClip:

El estudio MITRA-FR ^45^ evaluó pacientes con IMF severa e insuficiencia cardiaca en tratamiento médico, sometidos a reparación percutánea borde a borde versus terapia médica. Los resultados demostraron que no hubo diferencia significativa entre los grupos en mortalidad y rehospitalización a 12 meses. Por otra parte, el estudio COAPT ^(^^46^^)^ incluyó pacientes con IMF moderada a severa e insuficiencia cardiaca, comparando el uso de MitraClip más terapia médica óptima versus tratamiento médico aislado. Se observó que en el grupo de intervención (MitraClip) hubo disminución de hospitalización por falla cardíaca a los 24 meses (punto final primario), remodelamiento ventricular, muerte y hospitalización al año; necesidad de asistencia mecánica ventricular al año y muerte por todas las causas a los 24 meses; además, el número de pacientes a tratar (NNT) para prevenir una hospitalización dentro de los 24 meses de seguimiento fue 3,1 ^46^.

### d. Cirugía 

De acuerdo con la actualización del consenso de expertos del Colegio Americano de Cardiología 2020, la recomendación de tratamiento quirúrgico, asociado o no con revascularización miocárdica, es una postura que solo evidenció mejoría en calidad de vida, pero no en disminución de la mortalidad ^16^.

En casos seleccionados se puede optar por la reparación de la válvula mitral durante la revascularización miocárdica quirúrgica si los pacientes tienen enfermedad coronaria multiarterial más IMF moderada, aunque su beneficio en este contexto es incierto ^47^.

Si la IMF es severa, se podría plantear el tratamiento por reparación o reemplazo, si el paciente va a someterse a otra cirugía cardíaca, o como cirugía aislada si la clase funcional es avanzada a pesar de terapia médica óptima en ciertos casos. El modo de tratamiento va a depender de las condiciones del paciente, morfología del aparato valvular mitral y experiencia del centro ^1^^,^^6^.

La técnica quirúrgica de reparación versus reemplazo en IMF severa demostró mayor recurrencia de insuficiencia moderada-severa y mayores reingresos en el grupo de reparación valvular ^48^. Esto se puede deber a variables ecocardiograficas que empobrecen el éxito de la reparación, como el grado de deformación valvular y el remodelamiento del VI ^3^^,^^6^^,^^8^.

**Figura 3:**
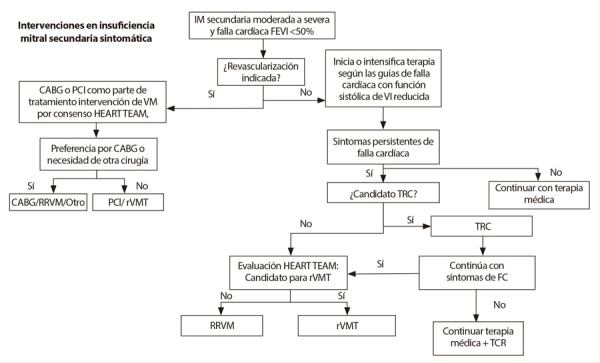
Algoritmo de manejo invasivo en insuficiencia mitral secundaria sintomatica IM: insuficiencia mitral, FEVI: fraccion de eyeccion del ventriculo izquierdo, CABG (ingles): bypass aorto-coronario, PCI: intervención percutanea coronario, VM: valvula mitral, RRVM: Reparacion o reemplazo de vlavula mitral, FC: falla cardiaca, TCR: terapia de resincronizacion cardiaca, rVMT: reparacion transacutanea de valvula mitral, VI: ventriculo izquierdo. Adaptado de Bonow RO, et al. 2020 Focused Update of the 2017 ACC Expert Consensus Decision Pathway on the Management of Mitral Regurgitation: A Report of the American College of Cardiology Solution Set Oversigth Committee in J Am Coll Cardio. 2020.

En caso de recurrencia, la decisión terapéutica es dada según el riesgo quirúrgico, probabilidad de buenos resultados y método quirúrgico previo. Esta debe ser una decisión multidisciplinaria basada en la experiencia del centro. Si el paciente es refractario a tratamiento médico y/o quirúrgico, y presenta un cuadro progresivo de insuficiencia cardiaca a estadios avanzados, se debe considerar la indicación de trasplante cardiaco si se cumplen los criterios necesarios para su aplicación ^1^^,^^6^^,^^49^**(**Figura 3**).**
